# Ethics and Privacy Implications of Using the Internet and Social Media to Recruit Participants for Health Research: A Privacy-by-Design Framework for Online Recruitment

**DOI:** 10.2196/jmir.7029

**Published:** 2017-04-06

**Authors:** Jacqueline Lorene Bender, Alaina B Cyr, Luk Arbuckle, Lorraine E Ferris

**Affiliations:** ^1^ Electronic Living Laboratory for Interdisciplinary Cancer Survivorship Research (ELLICSR) Health, Wellness, and Cancer Survivorship Centre Department of Supportive Care Princess Margaret Cancer Centre, University Health Network Toronto, ON Canada; ^2^ Dalla Lana School of Public Health University of Toronto Toronto, ON Canada; ^3^ Cancer Education Princess Margaret Cancer Centre University Health Network Toronto, ON Canada; ^4^ Electronic Health Information Lab Children's Hospital of Eastern Ontario (CHEO) Research Institute Ottawa, ON Canada

**Keywords:** Internet, social media, ethics, privacy, recruitment, cancer

## Abstract

**Background:**

The Internet and social media offer promising ways to improve the reach, efficiency, and effectiveness of recruitment efforts at a reasonable cost, but raise unique ethical dilemmas. We describe how we used social media to recruit cancer patients and family caregivers for a research study, the ethical issues we encountered, and the strategies we developed to address them.

**Objective:**

Drawing on the principles of Privacy by Design (PbD), a globally recognized standard for privacy protection, we aimed to develop a PbD framework for online health research recruitment.

**Methods:**

We proposed a focus group study on the dietary behaviors of cancer patients and their families, and the role of Web-based dietary self-management tools. Using an established blog on our hospital website, we proposed publishing a recruitment post and sharing the link on our Twitter and Facebook pages. The Research Ethics Board (REB) raised concern about the privacy risks associated with our recruitment strategy; by clicking on a recruitment post, an individual could inadvertently disclose personal health information to third-party companies engaged in tracking online behavior. The REB asked us to revise our social media recruitment strategy with the following questions in mind: (1) How will you inform users about the potential for privacy breaches and their implications? and (2) How will you protect users from privacy breaches or inadvertently sharing potentially identifying information about themselves?

**Results:**

Ethical guidelines recommend a proportionate approach to ethics assessment, which advocates for risk mitigation strategies that are proportional to the magnitude and probability of risks. We revised our social media recruitment strategy to inform users about privacy risks and to protect their privacy, while at the same time meeting our recruitment objectives. We provide a critical reflection of the perceived privacy risks associated with our social media recruitment strategy and the appropriateness of the risk mitigation strategies that we employed by assessing their alignment with PbD and by discussing the following: (1) What are the potential risks and who is at risk? (2) Is cancer considered “sensitive” personal information? (3) What is the probability of online disclosure of a cancer diagnosis in everyday life? and (4) What are the public’s expectations for privacy online and their views about online tracking, profiling, and targeting? We conclude with a PbD framework for online health research recruitment.

**Conclusions:**

Researchers, REBs, ethicists, students, and potential study participants are often unaware of the privacy risks of social media research recruitment and there is no official guidance. Our PbD framework for online health research recruitment is a resource for these wide audiences.

## Introduction

Increasingly, health researchers are turning to the Internet to recruit people for research studies [1-4]. The wide penetration of the Internet and the increasing use of social media (eg, wikis, blogs, online communities, and social networking sites) create many new avenues for research recruitment. In particular, social networking sites, such as Facebook, Twitter, and Google+, offer several potential advantages. These have considerable reach, providing access to large heterogeneous populations as well as small, hard-to-reach subpopulations dealing with sensitive, stigmatizing, or rare health conditions. They offer powerful sharing features that researchers can leverage to engage the public in spreading the word about a research project and recruitment by “liking,” “favoriting,” “replying to,” or “retweeting.” They are flexible; recruitment notices can be turned on and off and content can be changed in real time, allowing researchers the ability to control and evaluate recruitment efforts [5]. They are economical, reducing the time and effort involved in recruitment at reduced cost relative to other approaches [3].

However, the use of the Internet and social media as a health research recruitment tool raises unique ethical issues in part because personal and sensitive information may be collected from individuals without their knowledge or consent before they enroll in a study. The simple act of clicking on a recruitment notice is providing data to online behavioral advertising companies, leaving a potentially identifiable trail [6]. Online behavioral advertising (OBA) is a set of practices that companies engage in to track consumers’ online activities over time to deliver advertisements targeted to their inferred interests [7]. The problem is that many individuals either are unaware of the privacy risks of online activity or consciously accept a trade-off to their privacy [8]. For example, a man with sleep apnea was shocked to be followed by ads for such devices when he visited websites unrelated to the condition [8]. This man’s experience prompted an investigation by the Office of the Privacy Commissioner of Canada (OPC), which revealed that Google’s online advertising service used sensitive personal information about individuals’ online activities to deliver targeted health-related ads, which violates Canadian privacy law [9].

Although regulators like the OPC are mandated to enforce privacy laws, privacy breaches are not uncommon, and there is little guidance for researchers seeking to use social media for research recruitment. There are basic ethical principles, such as Respect for Persons, Concern for Welfare, and Justice, codified in the UN Declaration of Human Rights [10], the Nuremburg Code [11], the Declaration of Helsinki [12], and the Belmont Report [13]. There are general consensus statements, such as the Tri-Council Policy Statement (TCPS) [14] developed by Canada’s three federal funding agencies, that provide guidance on how to interpret and apply these basic ethical principles. For example, the TCPS explains that Respect for Persons can be achieved through “free, informed, and ongoing consent”; Concern for Welfare can be achieved by “minimizing risks and respecting and maintaining the welfare of participants,” which includes protecting their privacy; and Justice can be achieved by “treating all people fairly and equitably” [14]. In addition, the Ethics Working Committee of the Association of Internet Researchers (AoIR), an international professional association, has produced a set of guiding questions for researchers seeking to use the Internet for research [15]. However, these documents predate the Internet or social media, do not adequately address the unique ethical issues of social media as a recruitment tool, or do not offer practical solutions.

Many forms of Internet-based research could be considered ethically challenging because of the blurred public and private boundaries of online spaces [16], the dynamic and interactive nature of the media [17], and ease with which sensitive data can be accessed, shared, hacked, and/or replicated [18]. Online research recruitment introduces unique ethical issues because it may pose threats to the principles of Respect for Persons and Concern for Welfare in regard to privacy even before the consent to enroll in a study. Privacy is defined as an “individual’s right to be free from intrusion or interference by others” [14]. An important aspect of privacy is the right to control information about oneself. In the context of health research, this means that an individual should have the opportunity to exercise control over personal information by consenting to, or withholding consent for, the collection, use, and/or disclosure of information. Confidentiality, a related but distinct concept, refers to the obligation to “safeguard entrusted information from unauthorized access, use, disclosure, modification, loss, or theft” [14]. We [16], along with a handful of other researchers [19-23], explored the ethical and legal issues related to social media as a source of qualitative data, resulting in some recommendations. There is only one known study that explored the ethical aspects of social media as a recruitment tool. Curtis describes the ethical challenges of social networking and online recruitment for HIV research and concludes with a set of recommended best practices for HIV researchers [6].

Critical dialogue is needed to understand the pertinent ethical issues involved in online health research recruitment and the procedural solutions to protect the rights and safety of potential research participants. In this paper, we describe how we used the Internet and social media to recruit cancer patients and their family caregivers for a focus group study on dietary self-management behaviors, the ethical concerns raised by our institutional Research Ethics Board (REB), and the privacy-enhancing strategies we developed to address them. We include a critical reflection of the perceived privacy risks associated with our social media recruitment strategy and the appropriateness of the risk mitigation strategies that we employed by assessing their alignment with the principles of Privacy by Design (PbD) [24], a globally recognized standard for the protection of privacy [25]. We conclude by offering a PbD framework for online health research recruitment. While primarily directed at researchers, this framework for achieving PbD in online health research recruitment is intended to support and inform a wide array of stakeholders responsible for making decisions about the ethics of online health research recruitment.

## Methods

### Overview

We (JLB and ABC) explored the nutrition and culinary knowledge, attitudes, and behaviors of cancer patients and their family caregivers, and their views on Web-based tools to enhance dietary self-management behaviors. Lack of nutritional knowledge and culinary skills reduces the likelihood of practicing dietary self-management behaviors [26].

Initially, we relied on traditional recruitment methods, including posters placed at strategic locations (eg, elevators and clinics) in the hospital, in-person recruitment at our cooking and nutrition education classes, and targeted promotion of our study by email to our community partners. Despite this effort, these strategies did not help us reach our recruitment target and composition. Recruitment challenges are a persistent problem faced by researchers. A retrospective review of 404 clinical trials funded by two major funding agencies in the United Kingdom found that only 55% reached their recruitment target [27].

**Figure 1:**
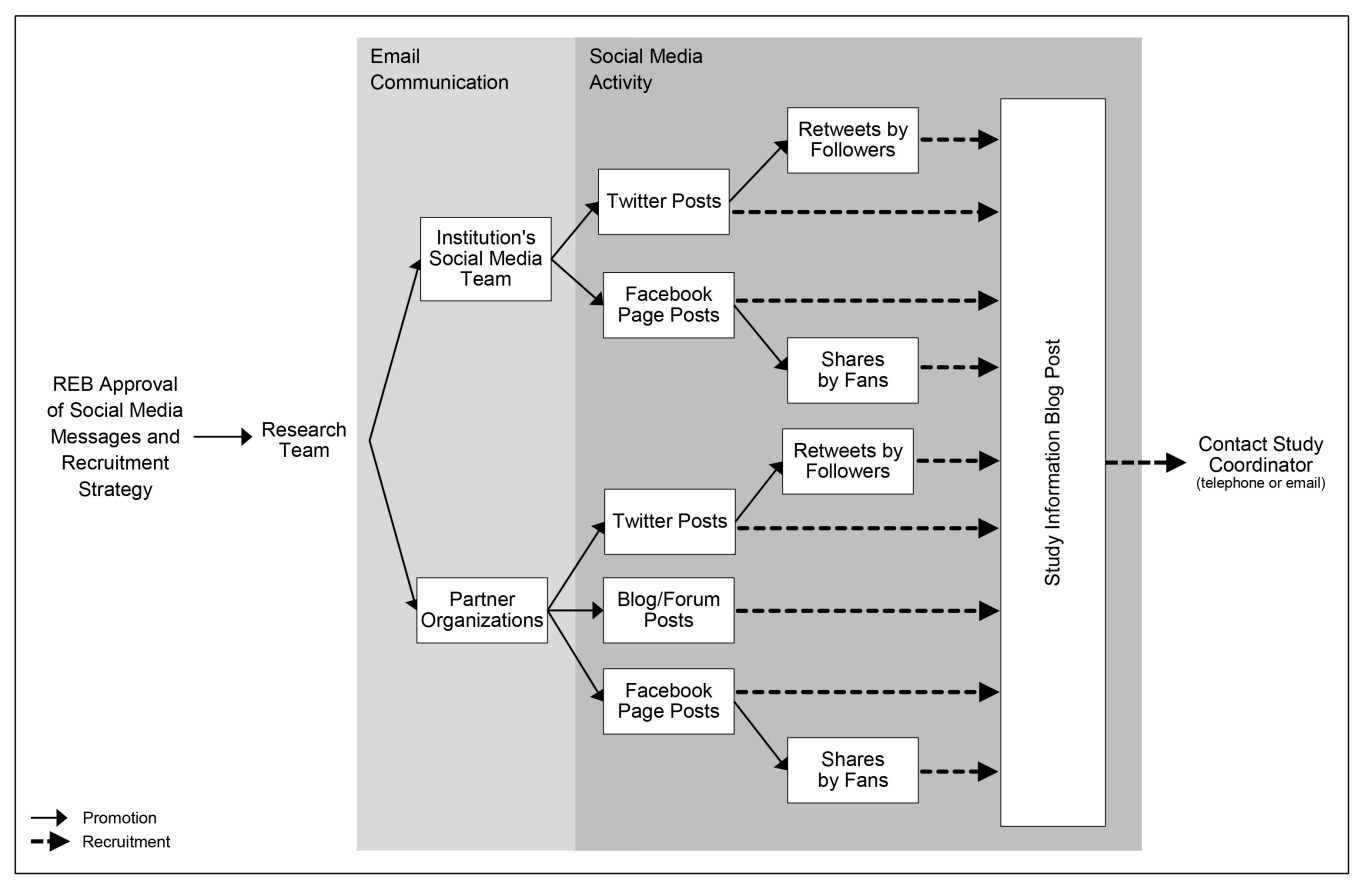
Initial social media recruitment strategy.

Encouraged by the evidence on the potential effectiveness of social media as a health research recruitment tool [1], we applied for institutional REB approval to use the Internet and social media to recruit study participants. Our social media recruitment strategy was multichannel (see Figure 1). We proposed to publish a recruitment notice on an established blog on our hospital website, share the link to the blog post on our Twitter and Facebook pages over 4 weeks, and ask our social media followers and community partners to share the link with their networks of connections.

### Ethical Concerns Raised by the Research Ethics Board

Our institutional REB raised concerns about the privacy risks associated with our proposed use of the Internet and social media for research recruitment. Specifically, they were concerned that by clicking on our social media recruitment messages (eg, “Seeking cancer patients for a study of nutrition and cooking”), individuals may unknowingly add personal and sensitive health information to their online profile, leaving an identifiable trail that may be used and disclosed by marketers.

The REB asked us to revise our social media recruitment strategy with the following questions in mind:

1. How will you inform users about the potential for privacy breaches and their implications?

2. How will you protect users from privacy breaches or inadvertently sharing potentially identifying information about themselves?

## Results

### Privacy-Enhanced Social Media Recruitment Strategy

#### Overview

Our revised social media recruitment strategy served to inform users about privacy risks and protect their privacy, while at the same time meeting our recruitment objectives. This *win-win* approach is a fundamental principle of PbD [24].

PbD was developed by the former Information and Privacy Commissioner of Ontario, Canada, Dr Ann Cavoukian in the late 1990s. It is an overarching framework for embedding privacy and data protection into information technologies, organizational processes, networked architectures, and entire systems of oversight in a credible and effective way [24,28]. It is based on the following seven foundational principles (verbatim): (1) Proactive not Reactive, Preventative not Remedial; (2) Privacy as the Default Setting; (3) Privacy Embedded into Design; (4) Full Functionality—Positive-Sum, not Zero-Sum; (5) End-to-End Security—Full Lifecycle Protection; (6) Visibility and Transparency—Keep it Open; and (7) Respect for User Privacy—Keep it User Centric [24].

In this section, we describe our revised social media recruitment strategy and reflect on the extent to which the privacy-enhancing measures that we used aligned with PbD. The principles of PbD and their descriptions are summarized verbatim in Table 1, along with our assessment of the extent to which our recruitment measures aligned with them.

**Table 1:** Applying the principles of Privacy by Design [24] to our case study^a^.

Principle	Short description	Alignment with Privacy by Design
1.Proactive not Reactive; Preventative not Remedial	PbD^b^ seeks to anticipate and prevent privacy-invasive events before they happen. PbD does not wait for privacy risks to materialize nor offer remedies after the fact.	Privacy notices proactively informed users about the privacy risks of social media, but required individuals to take action to protect their privacy. On the other hand, marketing headlines proactively protected individuals’ privacy by ensuring that those interested in the study were concealed within a broader population than just those targeted for recruitment. In contrast, editing or removing posts after publication represents a remedial, after-the-fact solution.
2.Privacy as the Default Setting	PbD seeks to deliver the maximum degree of privacy by ensuring that personal data are automatically protected. No action is required on the part of the individual to protect their privacy. It is built into the system, by default.	We built privacy protection into the recruitment strategy using marketing headlines and a hospital blog with a disabled comment feature to recruit interested individuals. Those that chose to enroll in the study did so through the hospital’s private data collection system without tracing back to social media.
3.Privacy Embedded into Design	PbD is embedded into the design and architecture of the system. It is not bolted on as an add-on, after the fact. Privacy is integral to the system, without diminishing functionality.	We embedded privacy into the design of the recruitment strategy using marketing headlines, without diminishing the functionality of social media. On the other hand, we lost functionality that could have enhanced the spread and exposure of our recruitment messages by opting to use a blog with a disabled comment feature and by proposing to edit and delete sensitive posts before publication.
4. Full Functionality —Positive-Sum, not Zero-Sum	PbD seeks to accommodate all legitimate interests and objectives in a positive-sum, win-win manner, not through a dated, zero-sum approach where unnecessary trade-offs are made.	Using marketing headlines is an example of a win-win, privacy-enhancing strategy. It increased the reach of the recruitment strategy (which one would expect to increase enrollment) without compromising privacy. Disabling the comment feature on the hospital blog, on the other hand, is not win-win because we traded function for privacy.
5. End-to-End Security —Full Lifecycle Protection	PbD explains that strong security measures are essential to PbD from start to finish. Embedding PbD into the system prior to the first element of information being collected ensures that all data are securely retained throughout the entire lifecycle of the data involved.	We used social media to garner interest in the research study, embedding privacy protection in the *consideration phase* well before enrollment, then used the hospital’s private and secure data collection system and procedures to protect interested and consenting study participants’ privacy and confidentiality from start to finish. However, we could have done a better job explaining our strategy to our community partners to ensure that they used it fully. We had no control over how the public responded to or shared our social media recruitment messages.
6.Visibility and Transparency —Keep it Open	PbD seeks to assure all stakeholders that whatever the business practice or technology involved, it is, in fact, operating according to the stated promises and objectives, subject to independent verification.	Our aim with privacy notices was two-fold: (1) to inform users about privacy risks and their implications; and (2) to be as open and transparent as possible. We also adhered to the procedural practices and requirements set by our governing bodies to protect the rights and safety of potential research participants. This included Research Ethics Board review of the research protocol and approval of all social media posts and privacy notices prior to publication.
7. Respect for User Privacy —Keep it User Centric	PbD requires architects and operators to keep the interests of the individual uppermost by offering such measures as strong privacy defaults, appropriate notice, and empowering user-friendly options.	We were cautious in our use of marketing headlines so as not to risk deceiving people or wasting their time. We used privacy notices to offer users appropriate notice and attempted to design them effectively, but we did not use a user-centered design approach to develop them nor did we test their effectiveness. In addition, we do not know people’s views on the marketing headline strategy. Some may have disliked the lack of directness in the notice to get them to the second site.

^a^The principles and their descriptions are described verbatim [24].

^b^PbD: Privacy by Design.

#### A. Inform About Privacy Risks With Privacy Notices

Providing notice and choice about data practices is an essential element of data protection frameworks like PbD [24]. Providing participants with enough information to adequately assess risks and potential benefits associated with their participation in research is a basic requirement of ethical research practice [14]. Privacy notices are a common strategy to make a system’s users aware of data practices involving personal information, which is supposed to enable users to make informed decisions [29]. If designed effectively, the notices can function to proactively alert the user about potential privacy risks and prompt them to take action to protect their privacy. Privacy notices can take many different forms, ranging from a privacy policy on a website, cookie consent notices shown in a banner on a webpage, to consumer warnings or permission notices in pop-up dialog boxes.

**Figure 2:**
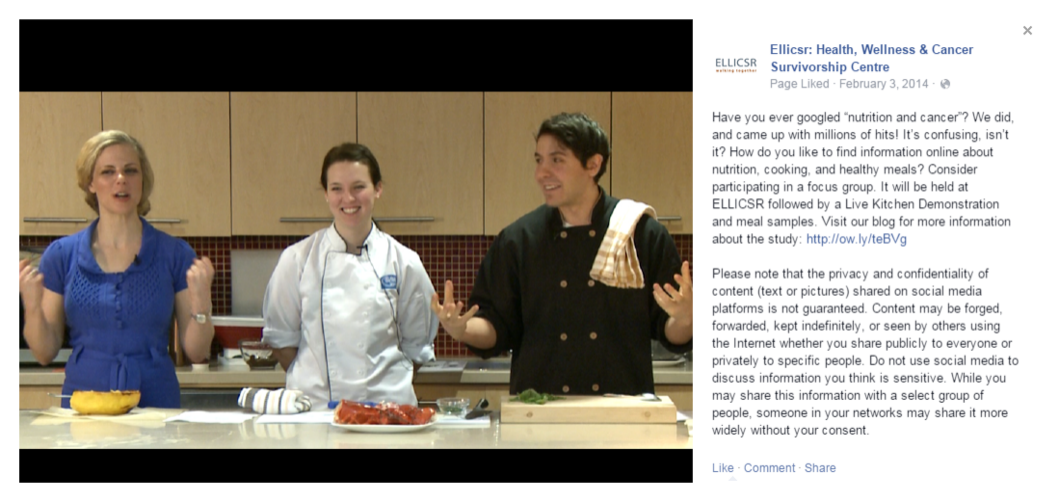
Facebook recruitment post with privacy disclaimer.

We developed privacy notices for the hospital blog and Facebook page and regularly tweeted disclaimers about the privacy risks of Twitter. We also included privacy notices in our email requests to community partners to spread the word about our research study. Privacy notices were written in plain language [30] and approved by a plain-language expert. Plain language is an evidence-based, patient-centered approach to writing health information. Plain language uses “familiar words, not jargon; active voice; and a conversational study to convey information clearly” [30]. All privacy notices were reviewed and approved by the REB before posting (see Table 2 and Figure 2).

**Table 2:** Privacy notices and disclaimers.

Medium	Privacy notice/disclaimer
Email	“Please note that the security of email messages is not guaranteed. Messages may be forged, forwarded, kept indefinitely, or seen by others using the Internet. Do not use email to discuss information you think is sensitive. Do not use email in an emergency since email may be delayed.”
Facebook	“Please also note that the privacy and confidentiality of content (text or pictures) shared on social media platforms is not guaranteed. Content may be forged, forwarded, kept indefinitely, or seen by others using the Internet whether you share publicly to everyone or privately to specific people. Do not use social media to discuss information you think is sensitive. While you may share this information with a select group of people, someone in your networks may share it more widely without your consent.”
Twitter	“The security of social media is not guaranteed. Contact us about the study. Don’t post if concerned about privacy.”^a^

^a^Please note that this tweet focuses on security as a possible threat to privacy if data is leaked. Privacy is not limited to security issues.

#### B. Protect Privacy Using Privacy-Enhanced Social Media Messages

We built privacy protection into our social media recruitment strategy using an Internet marketing approach known as *marketing headlines*. Internet marketing headlines aim to attract traffic by providing just enough information to make the reader curious, but not enough to satisfy their curiosity without clicking through. Marketing headlines are often associated with the less savory marketing practice *clickbait* that aims to trick people into following links online for the purpose of generating ad revenue [31]. In our case, we provided just enough information in our social media recruitment messages to attract the desired population, but not too much information that may cause them to inadvertently disclose personal health information through social media. Our goal was to garner public interest in our research while also attracting study participants.

For example, we originally proposed the following tweet to recruit participants for our study: “Seeking cancer patients for a study of nutrition and cooking @ELLICSRKitchen [URL].” Upon request by our REB, we removed the term “cancer patient” from all social media posts. The following is an example of a privacy-enhanced tweet: “Does #nutrition matter to you? Tell us what you think about #cooking and #cancer @ELLICSRKitchen [URL].”

This small change accomplished two goals: (1) it broadened the reach of our recruitment strategy by attracting a larger population of social media users; and (2) it protected patients’ privacy by default. Used in this way, marketing headlines is a win-win because we attract more interest in our work while pooling cancer patients we wish to recruit into a broader population of people interested in the subject of our research.

We asked our community partners to use our privacy-enhanced social media messages. All social media messages were reviewed and approved by a plain-language expert and the REB, and were published without modification.

#### C. Protect Privacy by Disabling Comment Feature or Moderating Comments

All social media messages included a link that directed interested individuals to the study recruitment notice on our hospital blog. At the time of publishing the recruitment notice on our hospital website, comments were not enabled on the blog platform due to hospital policy. Had commenting been enabled, we proposed to moderate any comments before they were made visible on the blog and remove references to potentially identifying or personal health information. While this strategy would have offered privacy protection, it does not represent a win-win because the blog software functionality was diminished to accomplish the privacy objectives. Allowing readers to freely post and share comments on the hospital blog could have generated online discussion about the study, which could have attracted more study participants, and represents a way to engage the public in spreading the word about a research project.

### Limitations

First, we used PbD to assess the appropriateness of our revised social media recruitment strategy *after the fact*. We encourage others to proactively use the PbD framework from the outset of the study design. Second, although our privacy notices were designed based on plain-language principles [30] and displayed prominently near the relevant contact information, it is possible that they were not seen or read. The evidence suggests that most privacy notices are not effective at informing consumers [32]. Based on a comprehensive review of research, Schaub et al offer best practices for improving the effectiveness of privacy notices [29]. These include the following: starting with a thorough understanding of a system’s information flows and data practices; tailoring notices to different audiences; providing concise, relevant, and actionable information; layering and contextualizing notices (eg, just-in-time notices without too much repetition to avoid habituation); and employing user-centered design to evaluate user attention, comprehension, and recall. Third, our social media recruitment strategy engaged other actors—our community partners and social media followers—to spread our social media recruitment messages. Although we provided our community partners and social media followers with privacy-enhanced social media messages to promote our study, we had no control over whether or how they adopted them or how the public responded to them, which could have resulted in advertent disclosures of personal information. Fourth, we do not know about the public’s views on marketing headlines as a research recruitment strategy. Some people may have disliked the lack of directness in the notice. Furthermore, we do not know the impact of this recruitment strategy on people who clicked but were ineligible to participate, and we do not know if eligible people found the recruitment strategy acceptable or if it negatively influenced their willingness to participate. Lastly, interested participants were required to contact the study coordinator by phone or email. A secure email form on the hospital webpage would have offered more privacy protection. Using a form controlled by our hospital server would ensure that the message was delivered to the intended recipient, with fewer chances of it being hacked from server to server, and that the message content is not scanned for keywords to trigger targeted ads, as is this case with Google email client [33].

## Discussion

### Were the Privacy Measures Appropriate?

#### Overview

Guidelines for the ethical conduct of human subject research state that risk mitigation strategies should be proportional to the magnitude and probability of risks involved [14]. Known as the proportionate approach, the level of risk posed by the research is used to determine “the level of review (eg, delegated or full board review), the approach to the actual review of the research itself, and the risk mitigation strategies required to protect the rights and safety of research subjects” [14]. This means that “the most intensive scrutiny, time, resources, and correspondingly, protection should be applied to the most ethically challenging research.” Similarly, PbD suggests that the strength of the privacy measure should match the sensitivity of the data [24].

We reflect on the perceived privacy risks associated with our social media recruitment strategy and the appropriateness of the risk mitigation strategies that we employed by discussing the following: (1) What are the potential risks and who is at risk? (2) Is cancer considered sensitive personal information? (3) What is the probability of online disclosure of a cancer diagnosis in everyday life? and (4) What are the public’s expectations for privacy online and their views about online tracking, profiling, and targeting?

#### A. What Are the Potential Risks and Who Is at Risk?

The primary risk associated with our recruitment strategy was the potential harm that a person may experience from the disclosure, collection, and use of personal and sensitive information—in this case a diagnosis of cancer—triggered by clicking on our social media recruitment messages. Potential harms associated with disclosure of health information like a cancer diagnosis could include stigmatization, discrimination, or damage to reputation, and may negatively affect relationships, job opportunities, and insurance options. However, we cannot assume that a person clicking on the recruitment message would experience these harms. What we do know is that they will likely receive advertising messages about cancer and/or eating well. It is possible that seeing such messages could be personally troubling for them, but we do not know if this is the case.

It is worth mentioning that there are documented cases of health data located in big data repositories or biobanks being repurposed by third parties for legal and security purposes. These unintended secondary uses of health data have included forensic investigations, civil lawsuits, border security, and identification of victims in mass casualty events [34]. For a thorough discussion of documented and hypothetical secondary uses of online health data collections, see O’Doherty et al [34].

In terms of who is at risk, it cannot be assumed that the person clicking on the recruitment message was revealing information about himself or herself at all. Spouses, children, siblings, other family members, and friends play a vital role in searching for health information. Research conducted by the Pew Research Center indicates that half of online health information research is on behalf of someone else [35]. Furthermore, as our recruitment blog post explained, we were seeking cancer patients and their family caregivers to participate in our research. Hence, if a caregiver clicked on our social media recruitment message, he or she would not have been revealing information about himself/herself, which was presumed to be the case in the ethics review. However, these individuals could have still received advertising messages about cancer and/or eating well, which they may or may not have found troubling.

#### B. Is Cancer “Sensitive” Personal Information, Requiring More Privacy Protection?

The Canadian Personal Information Protection and Electronic Documents Act (PIPEDA) defines personal information as “information about an identifiable individual” [36]. The OPC takes the position that information involved in online tracking and targeting constitutes personal information [7]. Principle 3 of PIPEDA states that “the knowledge and consent of the individual are required for the collection, use, or disclosure of personal information, except where inappropriate” [36]. Furthermore, the privacy act goes on to explain that “organizations must obtain an individual’s consent for all disclosures of their personal information to any third party unless one of PIPEDA’s exceptions to consent can be applied.” The magnitude or seriousness of harms associated with the disclosure of personal information depends, in part, on whether the information is considered “sensitive.” According to PIPEDA, some information is almost always considered to be sensitive (eg, medical records and income records); however, any information can be sensitive depending on the context. The US National Committee on Vital and Health Statistics (NCVHS) has done some further work defining sensitive health information, which they explain carries unusually high risks in the event of disclosure [37]. Based on public consultations and expert deliberation, categories of health information considered sensitive by the NCVHS include those related to domestic violence, genetics, mental health, reproductive care, and substance abuse [37]—not cancer. That being said, as explained in PIPEDA, sensitivity is subjective and depends on the individual’s circumstances, and the context in which the information is shared [36]. The Google health ads case is evidence of this as sleep apnea was considered sensitive personal information [9]. The main point is that health information is considered sensitive personal information, but within health information, there are gradients of sensitivity and cancer may be considered less sensitive personal health information.

#### C. What Is the Probability of the Risks and Harms Occurring in Everyday Life?

It is highly probable that cancer patients who clicked on our social media recruitment messages already disclosed their cancer diagnosis online, thereby exposing themselves to related harms. First, the majority of cancer patients report using the Internet as a source of health information. For example, 86% of a sample of 202 thyroid cancer patients [38] surveyed from the same hospital where this study was conducted, and 68% of a sample of 824 Canadian prostate cancer patients [39], reported using the Internet to search for information related to their cancer. As people spend more time online, they leave a digital trail. Second, given the scope and scale of information collected by third-party advertisers and the sophisticated means of collecting and analyzing disparate pieces of data [7], it is reasonable to assume that Internet search queries about cancer could be linked to an individual. Typical information collected in Internet log files includes the following: IP address, pages visited, length of time spent on pages, advertisements viewed, articles read, purchases made, search terms or other information entered on a site, user preferences such as language, operating system, and geographical location [8]. Additional data may be gathered from social networking sites where individuals volunteer significant amounts of personal information. Third, we used our departmental Facebook and Twitter pages to promote our research study to our social media followers. The people who follow us on social media have likely already “disclosed” to third-party trackers that, at the very least, they are interested in cancer by choosing to follow a social media account affiliated with a cancer center. Therefore, using our marketing headline approach, we would not subject cancer patients to disclose more than an interest in cancer, which they likely have already provided online.

#### D. What Are the Public’s Privacy Expectations and Views on Online Behavioral Advertising?

A total of 90% of Canadians are concerned about the privacy impact of new technologies and 98% want strong privacy laws [8]. People between the ages of 45 and 65 years are more likely to express high levels of concern about the privacy impact of new technologies than those 25 and under [34]. However, teenage social media users seem to care more about online privacy when it comes to their personal health information. Motivated by a need for self-protection as a chronically ill patient and self-definition as a regular teenager, a qualitative study (N=20) revealed that Canadian teenagers (12-18 years old) with a chronic illness were selective about sharing personal health information on social media [40]. In general, teenagers are less concerned about the collection of personal information by governments and companies, but very concerned about their social privacy, or having control over the content of their interactions with others [40]. When it comes to OBA, 50% of Canadians surveyed in 2009 were “somewhat uncomfortable” with tracking-based advertising [8]. However, a 2011 report by KPMG consulting firm revealed that 46% of Canadians were “somewhat willing” to have their online usage tracked by advertisers if there are benefits [41]. Benefits of OBA for the consumer include free online content, more relevant advertising, and enhanced browsing experience [41]. A population-based telephone survey of Americans suggests that consumers would be more willing to accept OBA if there was more transparency, consumer choice, and data retention limits [42]. Complicating a clear understanding of the public’s views and expectations with regard to online privacy is the well-known privacy paradox [43]. Most people would say that they care about their online privacy but do not act on that concern, revealing increasing amounts of personal information that can be used and disclosed by governments and marketers [43].

### Disparate Norms Within and Across Disciplines and Research Ethics Boards

A further challenge for researchers seeking to use the Internet and social media for research recruitment is the disparate norms about what is and what is not ethical across research communities. Researchers are guided by different disciplinary methodological approaches, norms, and conventions, and regulations for ethical online research vary across disciplines. What is considered ethically acceptable in one discipline may not be in another [44]. The same holds true for different REBs. Moreover, the same REB may reach different conclusions about the same technological approach across studies. Nebeker and colleagues show that visual imaging and location-tracking devices (eg, Global Positioning System) are reviewed inconsistently in one institution [45]. While research plans incorporated consistent descriptions of each device and associated potential risks, REB letters revealed inconsistent perceptions of potential study risks associated with the collocation of location data should a data breach occur [45]. Inconsistent perceptions about the potential risks involved in research that uses new technologies like social media make the REB protocol development and review process challenging for researchers. However, researchers are not the only ones grappling with the unique ethical issues of online research. REBs may be unfamiliar with these new technologies, prompting confusion about what actions are necessary and appropriate to effectively evaluate and mitigate potential risks. Furthermore, there may also be some differences in where different REBs draw the line between participant autonomy versus participant protection.

### Privacy by Design for Online Health Research Recruitment

We have shown that PbD is a useful framework for designing, evaluating, and achieving privacy in online health research recruitment. Applying the principles of PbD helped to identify the privacy strengths, weaknesses, and gaps in our recruitment strategy. Based on alignment with PbD principles, use of marketing headlines was the strongest privacy measure used whereas privacy notices were the weakest. Contrary to the principles of PbD, we made trade-offs in favor of privacy protection, such as agreeing to disable the comment functionality on the hospital blog, which traded function for privacy. PbD also alerted us to areas in need of improvement, such as the privacy gaps created by engaging others in implementing our recruitment strategy. To fully embed privacy into the design of a recruitment strategy, all parties involved in implementing it should endorse the PbD approach.

By applying PbD, we also identified areas in need of further research. While PbD is becoming the standard for privacy protection in many jurisdictions around the world [28], there is little practical guidance on how to apply the seven foundation principles [46]. For example, transparency and empowering user-centered options are key principles of PbD, but the framework provides little practical guidance on how to effectively design privacy notices using these principles. Schaub et al’s compilation of best practices for privacy notices is an excellent complementary resource in this regard [29]. As a first step, we need a better understanding of the public’s views on the privacy risks of online health research recruitment and Web-based research, including the probability and magnitude of harm as well as what privacy protection would be appropriate or may create potential barriers to access. In parallel, further research is needed to understand how to effectively design strong privacy defaults, appropriate notice mechanisms, and empowering options, and to examine the impact of these privacy measures on the public’s online behaviors, including participation in health research studies. In this study, we did not consider informing users about the various strategies to protect their online privacy, but we think this is important. Future research should consider designing and evaluating educational efforts to teach patients and their families about these strategies. These strategies include the following: clearing your Internet browser history (eg, cookies); installing Internet browser extensions that block ads, or that reveal and block the websites that track your browser history; or using InPrivate Browsing to stop the computer from tracking your website history.

Another privacy tool, Privacy Impact Assessment (PIA), deserves mention. PIAs aim to “identify the potential privacy risks of new or redesigned programs and to eliminate or reduce those risks to an acceptable level” [46]. They are generally used to ensure that an organization is complying with legislative and regulatory requirements. PIAs may be useful tools to consider for the assessment of the privacy risks of an online health research recruitment strategy. However, typical PIAs are not grounded in the PbD framework and they do not provide overarching principles to guide the design and implementation of privacy protection. Jeselon and Fineberg recommend using the PbD framework to augment PIAs to achieve a more holistic approach to privacy protection and offer practical guidance on how to apply PbD to PIAs [47].

Based on our experiences with this case study, we offer a PbD framework for online health research recruitment. We drew on the principles of PbD [24] and examples of its application [28,47], as well as recommendations from the AoIR [15] and the Secretary’s Advisory Committee on Human Research Protections [18]. In this framework, we offer a set of privacy questions and considerations to guide the ethical design and conduct of studies that use the Internet and social media as a health research recruitment tool. We describe the principles, guiding questions, and application considerations of this framework in Table 3. The PbD principles are verbatim. We have drawn on recommendations from cited sources to aid the reader in their application.

**Table 3:** Privacy by Design framework for online health research recruitment: Proposed considerations for researchers and institutional Research Ethics Boards.

Privacy-by-Design principles		Considerations
**A. Consider the nature of the study, the target population, and the sensitivity of the data**		
	Justification	Why is it necessary to use the Internet and social media to recruit participants for your research project?
	Context	Where does the study recruitment take place? What are the terms of use and privacy policies of the recruitment sites or applications? What are users’ privacy expectations regarding the recruitment sites or applications?
	Sensitivity	What is the subject of study? Is the data considered personal information? Is the data considered “sensitive” personal information? What are the privacy expectations commonly associated with these types of data?
	Vulnerability	Who are the recruitment targets? What additional privacy measures may be required to protect the privacy of vulnerable individuals?
**B. Apply Privacy by Design [24]**		
	Proactive not Reactive; Preventative not Remedial	What are the potential privacy risks and related harms associated with the recruitment strategy? Do certain data, people, or groups require more privacy protection? PbD^a^ Application: Anticipate and prevent privacy-invasive events before they happen—before individuals are even exposed to the recruitment strategy—as opposed to offering remedies for resolving privacy breaches once they have occurred [24]. Adopt and implement strong privacy practices early and continuously, and use systematic methods to recognize and correct weakening links, privacy measures, or data protection practices before privacy risks occur [28].
	Privacy as the Default Setting	If an individual does nothing, is their privacy still intact when they are exposed to the recruitment strategy or do they have to take action (eg, opt out or add a privacy measure) to protect their privacy? PbD Application: Aim to deliver the maximum degree of privacy by ensuring that personal data are automatically protected without the individual having to do anything to protect their privacy [47]. Keep the collection of personal information to a minimum, justify additional data collection on a data-by-data basis, and use default settings of technologies that offer the most privacy protection [28].
	Privacy Embedded into Design	Is your privacy-enhancing measure built into the design of your recruitment strategy or has it been bolted on as an add-on, after the fact? PbD Application: Make privacy a core component of your recruitment strategy from the outset of the study, so that it is an essential component of the study design [47]. Embed privacy into the recruitment technologies, operations, and information architectures in a holistic, integrative way [28].
	Full Functionality—Positive-Sum not Zero-Sum	Does your recruitment strategy offer privacy protection without sacrificing your recruitment goals and objectives? PbD Application: Consider all legitimate interests and objectives of the recruitment strategy and aim to accommodate them in optimal ways to ensure the individual’s privacy is protected without any unnecessary trade-offs between privacy and functionality, security, or your recruitment goals [47]. Select privacy-enhancing measures that help to achieve your recruitment goals, maintaining full functionality and full security while protecting privacy [28].
	End-to-End Security—Full Lifecycle Protection	Are there any weak links or gaps in the implementation or oversight of your recruitment strategy? PbD Application: Consider how information, particularly personal information, will flow and be accessed, and by whom, throughout the entire lifecycle of the study. Embed privacy-enhancing measures and data security measures into the recruitment strategy before the first element of data is collected by you as a researcher or by third parties, and extend that security in a comprehensive and systematic manner throughout the entire lifecycle of the data involved [28].
	Visibility and Transparency—Keep it Open	Are all people and organizations involved in recruiting participants (directly or indirectly) operating according to stated promises and objectives, and is information about their privacy policies and practices readily available to the public? PbD Application: Ensure that all recruitment actors are operating according to their stated privacy practices (eg, policies and procedures related to the collection, use, and storage of personal information) and that these are made visible and transparent to enable users to make an informed choice about whether to participate in the study or not [47]. When sharing study information with collaborators or third parties, ensure that they use equivalent data protection measures through contractual processes or others means [28].
	Respect for User Privacy—Keep it User Centric	Are your privacy measures user centric? Have they been designed with the user in mind? Are they simple to use and written in easy-to-understand plain language? Have they been tested and approved by users? PbD Application: Respect for User Privacy is at the heart of PbD. Use user-centered and empowering, user-friendly, privacy-enhancing recruitment technologies, policies, and procedures so that individuals can exercise their privacy rights and make informed privacy decisions [28]. As explained by Dr Cavoukian, “The most privacy-enhancing solutions and results are usually those that are consciously designed around the interests, needs, and expectations of individuals and users, who typically have the greatest vested interest in the management of their personal data by others” [28].

^a^PbD: Privacy by Design.

### Conclusions

Researchers, REBs, ethicists, students, and potential study participants are often unaware of the privacy risks of Internet and social media health research recruitment and there is no official guidance. From this case study, some may conclude that the REB’s perceptions of the potential risks involved in our research study and our revised privacy-enhanced recruitment strategy did not match the magnitude and probability of the risks involved. On the other hand, others may argue that given that hospitals occupy an important trust relationship with patients and the public, hospital REBs should apply the precautionary principle as their use of social media may provide a false sense of security. We have shown that PbD is a useful framework for designing, evaluating, and achieving privacy in Web-based research recruitment. We offer our PbD framework for online health research recruitment for researchers and REBs to guide the ethical design, review, and conduct of studies that use the Internet and social media as a health research recruitment tool. Future research should focus on designing effective privacy notices and measures and evaluating their impact.

## Abbreviations

AoIR: Association of Internet Researchers
CHEO: Children's Hospital of Eastern Ontario
ELLICSR: Electronic Living Laboratory for Interdisciplinary Cancer Survivorship Research
NCVHS: National Committee on Vital and Health Statistics
OBA: online behavioral advertising
OPC: Office of the Privacy Commissioner of Canada
PbD: Privacy by Design
PIA: Privacy Impact Assessment
PIPEDA: Personal Information Protection and Electronic Documents Act
REB: Research Ethics Board
TCPS: Tri-Council Policy Statement

## References

[1] Alshaikh F, Ramzan F, Rawaf S, Majeed A (2014). Social network sites as a mode to collect health data: A systematic review. J Med Internet Res.

[2] Reaves AC, Bianchi DW (2013). The role of social networking sites in medical genetics research. Am J Med Genet A.

[3] Park BK, Calamaro C (2013). A systematic review of social networking sites: Innovative platforms for health research targeting adolescents and young adults. J Nurs Scholarsh.

[4] Topolovec-Vranic J, Natarajan K (2016). The use of social media in recruitment for medical research studies: A scoping review. J Med Internet Res.

[5] Frandsen M, Walters J, Ferguson SG (2014). Exploring the viability of using online social media advertising as a recruitment method for smoking cessation clinical trials. Nicotine Tob Res.

[6] Curtis BL (2014). Social networking and online recruiting for HIV research: Ethical challenges. J Empir Res Hum Res Ethics.

[7] (2015). Office of the Privacy Commissioner of Canada.

[8] (2011). Office of the Privacy Commissioner of Canada.

[9] (2015). Office of the Privacy Commissioner of Canada.

[10] (1948). The Universal Declaration of Human Rights.

[11] (1949). Trials of War Criminals Before the Nuremberg Military Tribunals Under Control Council Law, No. 10.

[12] World Medical Association (2013). Declaration of Helsinki: Ethical Principles for Research Involving Human Subjects.

[13] The National Commission for the Protection of Human Subjects of Biomedical and Behavioral Research (1979). Office for Human Research Protections.

[14] (2014). Tri-Council Policy Statement: Ethical Conduct for Research Involving Humans.

[15] Markham A, Buchanan E (2012). Ethical Decision-Making and Internet Research: Recommendations From the AoIR Ethics Working Committee (Version 2.0).

[16] Bender J, Norman C, Jadad A (2010). Negotiating consent in the Facebook era: Insights from research on online health communities with youth. Proceedings of the ACM Conference on Computer Supported Cooperative Work (CSCW).

[17] O'Grady L, Witteman H, Bender JL, Urowitz S, Wiljer D, Jadad AR (2009). Measuring the impact of a moving target: Towards a dynamic framework for evaluating collaborative adaptive interactive technologies. J Med Internet Res.

[18] Office for Human Research Protections (2013). Considerations and Recommendations Concerning Internet Research and Human Subject Research Regulations, With Revisions.

[19] Moreno MA, Fost NC, Christakis DA (2008). Research ethics in the MySpace era. Pediatrics.

[20] Flicker S, Haans D, Skinner H (2004). Ethical dilemmas in research on Internet communities. Qual Health Res.

[21] Eysenbach G, Till JE (2001). Ethical issues in qualitative research on Internet communities. BMJ.

[22] Bond CS, Ahmed OH, Hind M, Thomas B, Hewitt-Taylor J (2013). The conceptual and practical ethical dilemmas of using health discussion board posts as research data. J Med Internet Res.

[23] Zimmer M (2010). “But the data is already public”: On the ethics of research in Facebook. Ethics Inf Technol.

[24] Cavoukian A (2011). Privacy by Design: The 7 Foundational Principles.

[25] Unknown (2010). Resolution on privacy by design. Proceedings of the 32nd International Conference on Data Protection and Privacy Commissioners.

[26] Garcia AL, Vargas E, Lam PS, Shennan DB, Smith F, Parrett A (2014). Evaluation of a cooking skills programme in parents of young children--A longitudinal study. Public Health Nutr.

[27] Sully B, Julious S, Nicholl J (2013). A reinvestigation of recruitment to randomised, controlled, multicenter trials: A review of trials funded by two UK funding agencies. Trials.

[28] Cavoukian A, Gutwirth S, Leenes R, de Hert P, Poullet Y (2013). Privacy by Design: Leadership, methods, and results. European Data Protection: Coming of Age.

[29] Schaub F, Balebako R, Durity A, Cranor L (2015). A design space for effective privacy notices. Proceedings of the Symposium on Usable Privacy and Security (SOUPS).

[30] Wizowski L, Harper T, Hutchings T (2014). Writing Health Information for Patients and Families: A Guide to Developing Patient Education Materials That Promote Health Literacy. 4th edition.

[31] English Oxford Living Dictionaries.

[32] Cranor L (2012). Necessary but not sufficient: Standardizing mechanisms for privacy notice and choice. J Telecommun High Technol Law.

[33] Schofield J (2013). The Guardian.

[34] O'Doherty KC, Christofides E, Yen J, Bentzen H, Burke W, Hallowell N, Koenig BA, Willison DJ (2016). If you build it, they will come: Unintended future uses of organised health data collections. BMC Med Ethics.

[35] Fox S, Duggan M (2013). Pew Research Center.

[36] (2015). Personal Information Protection and Electronic Documents Act.

[37] (2010). Recommendations Regarding Sensitive Health Information.

[38] Bender JL, Wiljer D, Sawka AM, Tsang R, Alkazaz N, Brierley JD (2016). Thyroid cancer survivors' perceptions of survivorship care follow-up options: A cross-sectional, mixed-methods survey. Support Care Cancer.

[39] Bender JL, Feldman-Stewart D, Tong C, Pai H, Au JWY, Brundage MD, Robinson JW, Davison BJ, Kazanjian A (2015). Prostate cancer patients information technology use, preferences and needs: A cross-sectional population based survey. Proceedings of the Society of Behavioral Medicine 38th Annual Meeting and Scientific Sessions.

[40] van der Velden M, El Emam K (2013). “Not all my friends need to know”: A qualitative study of teenage patients, privacy, and social media. J Am Med Inform Assoc.

[41] (2012). The Converged Lifestyle: Consumers and Convergence 5.

[42] Turow JM, King J, Hoofnagle C, Bleakley A, Hennessey M (2009). Social Science Research Network (SSRN).

[43] Radin TJ (2001). The privacy paradox: E-commerce and personal information on the Internet. Bus Prof Ethics J.

[44] Bos N, Karahalios K, Musgrove-Chávez M, Poole E, Thomas J, Yardi S (2009). Research ethics in the Facebook era: Privacy, anonymity, and oversight. Proceedings of the Conference on Human Factors in Computing Systems (CHI).

[45] Nebeker C, Linares-Orozco R, Crist K (2015). A multi-case study of research using mobile imaging, sensing and tracking tehnologies to objectively measure behavior. J Res Adm.

[46] (2016). Office of the Privacy Commissioner of Canada.

[47] Jeselon P, Fineberg A (2011). A Foundational Framework for a Privacy by Design Privacy Impact Assessment.

